# Patterns of alcohol and drug utilization in trauma patients during the COVID-19 pandemic at six trauma centers

**DOI:** 10.1186/s40621-021-00322-0

**Published:** 2021-03-22

**Authors:** Constance McGraw, Kristin Salottolo, Matthew Carrick, Mark Lieser, Robert Madayag, Gina Berg, Kaysie Banton, David Hamilton, David Bar-Or

**Affiliations:** 1 Trauma Research Department, Injury Outcomes Network, Englewood, CO USA; 2 Trauma Services Department, Medical City Plano, Plano, TX USA; 3 Trauma Services Department, Research Medical Center, Kansas City, MO USA; 4 Trauma Services Department, St. Anthony Hospital, Lakewood, CO USA; 5 Trauma Services Department, Wesley Medical Center, Wichita, KS USA; 6 Trauma Services Department, Swedish Medical Center, Englewood, CO USA; 7 Trauma Services Department, Penrose-St. Francis Health Services, Colorado Springs, CO USA

**Keywords:** Alcohol abuse, Substance abuse, COVID-19, Substance use disorder, Traumatic injuries

## Abstract

**Background:**

Since the national stay-at-home order for COVID-19 was implemented, clinicians and public health authorities worldwide have expressed growing concern about the potential repercussions of drug and alcohol use due to social restrictions. We explored the impact of the national stay-at-home orders on alcohol or drug use and screenings among trauma admissions.

**Methods:**

This was a retrospective cohort study at six Level I trauma centers across four states. Patients admitted during the period after the onset of the COVID-19 restrictions (defined as March 16, 2020-May 31, 2020) were compared with those admitted during the same time period in 2019. We compared 1) rate of urine drug screens and blood alcohol screens; 2) rate of positivity for drugs or alcohol (blood alcohol concentration ≥ 10 mg/dL); 3) characteristics of patients who were positive for drug or alcohol, by period using chi-squared tests or Fisher’s exact tests, as appropriate. Two-tailed tests with an alpha of *p* < 0.05 was used on all tests.

**Results:**

There were 4762 trauma admissions across the study period; 2602 (55%) in 2019 and 2160 (45%) in 2020. From 2019 to 2020, there were statistically significant increases in alcohol screens (34% vs. 37%, *p* = 0.03) and drug screens (21% vs. 26%, *p* < 0.001). Overall, the rate of alcohol positive patients significantly increased from 2019 to 2020 (32% vs. 39%, *p* = 0.007), while the rate of drug positive patients was unchanged (57% vs. 52%, *p* = 0.13). Of the 1025 (22%) patients who were positive for alcohol or drugs, there were significant increases in a history of alcoholism (41% vs. 26%, p < 0.001), and substance abuse (11% vs. 23%, p < 0.001) in the 2020 period. No other statistically significant differences were identified among alcohol or drug positive patients during COVID-19 compared to the same period in 2019.

**Conclusions:**

Our first wave of COVID-19 data suggests that trauma centers were admitting significantly more patients who were alcohol positive, as well those with substance use disorders, potentially due to the impact of social restrictions and guidelines. Further longitudinal research is warranted to assess the alcohol and drug positive rates of trauma patients over the COVID-19 pandemic.

## Background

On January 20, 2020, the first case of COVID-19 was reported in the United States in an individual who had returned from a trip to visit family in Wuhan, China. (Chan et al. 2020; Shereen et al. 2020) Because of the rapid spread of the virus and the severity of the illness caused by it, public health officials have driven sweeping reforms to stem further dissemination of SARSCoV-2. On March 16, 2020, the White House announced a nationwide “social distancing” order to remain in place for a minimum of 15 days; (NPR 2020) social distancing recommendations were subsequently extended until April 30, 2020. (CNN 2020) Subsequently, individual states began to issue stay-at-home or shelter-in-place orders as well. (Kaiser Family Foundation 2020) To date (December 2020), state mandates to minimize transmission of the virus persist, including but not limited to mandatory face masks in public places, social distancing of six feet, and varying restrictions on size of social gatherings. (AARP 2020).

Perhaps as a consequence of these efforts, there are likely to be a number of unforeseeable implications due to psychological distress triggered by financial difficulties, social isolation, and uncertainty about the future. Studies have shown that social isolation and loneliness are associated with alcohol and drug abuse and furthermore, previous health-related disasters have led to posttraumatic stress disorder (PTSD) and alcohol dependence. (Reynolds et al. 2008; Sprang and Silman 2013; Taylor et al. 2008; Wu et al. 2009; Wu et al. 2008) Among 1074 people surveyed from Hubei and other provinces during the COVID-19 pandemic, hazardous drinking increased and alcohol dependency reached 1.6% among young people (aged 10–41), (Ahmed et al. 2020) while in the United Kingdom the number of high risk drinkers has almost doubled during lockdown. (The Telegraph 2020). Consequences also apply to substance users who, as a result of the COVID-19 restrictions, may no longer have access, potentially resulting in alcohol and drug withdrawal complications. (Columb et al. 2020; Rehm et al. 2020) Although numerous studies indicate the potential for a perfect storm between substance use disorders (SUDs) and COVID-19, (Columb et al. 2020; Narasimha et al. 2020; Satre et al. 2020; Spagnolo et al. 2020) there is currently a dearth of literature on how this applies to the US healthcare system. Particularly, traumatic injuries are still occurring during the lockdown, but there are minimal patient data examining alcohol or drug use during COVID-19 within trauma centers.

This study examines the effect of the current COVID-19 pandemic on 1) the rate of screening for alcohol and drugs, 2) the rate of positivity for alcohol or drugs, and 3) characteristics of alcohol or drug positive patients.

## Methods

This retrospective cohort study was performed by the Injury Outcomes Network, a collaborative research network of six community based, American College of Surgeons verified Level I trauma centers: three trauma centers are located in Colorado (Swedish Medical Center, St. Anthony Hospital, Penrose Hospital), and three trauma centers are located outside of Colorado (Medical City Plano, Plano, TX, Research Medical Center, Kansas City, MO, and Wesley Medical Center, Wichita, KS). The study included trauma patients ≥18 years, admitted from March 16, 2019-May 31, 2020. Patients were compared between two admission time periods: Period 1 (pre-COVID-19, 3/16/2019–5/31/2019); and Period 2 (COVID-19 with social distancing, 3/16/2020–5/31/2020). The same dates in 2019 were used as a pre-COVID-19 control group due to potential seasonal variation in the characteristics of patients with traumatic injury. All study data were collected from facilities’ trauma registry. This study was approved by institutional review boards at each of the participating centers.

Outcomes included: the proportion of patients with a blood alcohol screen (performed by direct blood tests ≤24 h of first hospital encounter) as well as a positive blood alcohol concentration (BAC, ≥ 10 mg/deciliter: the smallest alcohol positive cut-point across all hospitals); the proportion of patients with a standard multi-drug urine drug screen (UDS) panel (performed ≤24 h of first hospital encounter) as well as a positive result for any of the following: amphetamines, methamphetamine, barbiturates, benzodiazepines, cocaine, opiates, Phenylcyclohexyl piperidine (PCP), Methylenedioxy-methylamphetamine (ecstasy), and marijuana (tetrahydrocannabinol [THC]).

The following covariates were collected on each patient: sex, age, race, injury severity score (ISS, 1-9, 10-15, ≥16), hospital length of stay (LOS), ICU stay (yes/no), cause of injury (fall, assault, gunshot wound (GSW), motor vehicle crash (MVC), motorcycle crash (MCC), other), the presence of the following comorbid conditions: mental illness, smoking, alcoholism, substance abuse (alcoholism and substance abuse together referred to as substance use disorders (SUDs)); and subsequent complications such as alcohol withdrawal syndrome. Alcoholism and substance abuse must be present prior to injury and are both consistently defined in the National Trauma Databank Data Dictionary according to the American Psychiatric Association DSM 5, 2013 definitions for alcohol and substance use disorders (American College of Surgeons. Committee on Trauma 2020). Comorbidities were also evaluated using the Charlson Comorbidity Index (CCI), which assigns patients a score based on age and specific chronic comorbidities and aims to predict a patient’s risk of mortality post-hospitalization. The CCI is also frequently used as a measure of a patient’s overall comorbidity burden. (Austin et al. 2015; Brusselaers and Lagergren 2017; NCI Comorbidity Index overview, 2019).

### Statistical analyses

The number of patients screened and the number who were positive for alcohol or drugs were examined as percent (n) by COVID-19 time period, to determine whether the composition of alcohol/drug positive patients changed during the pandemic. χ^2^ tests and Fisher’s exact tests were used for categorical variables, while continuous data was analyzed using Wilcoxon Mann-Whitney U tests and Kruskal Wallis tests, as necessary. A significance level of α = 0.05 and SAS 9.4 were used to conduct all statistical analyses.

## Results

There were 4762 trauma patients admitted across the study period: 55% in Period 1 (pre-COVID-19) and 45% in Period 2 (COVID-19 with social distancing). From Period 1 to Period 2, there were statistically significant increases in alcohol screens (34% vs. 37%, *p* = 0.03, Table 1), drug screens (21% vs. 26%, *p* = 0.001), the number screened for both alcohol and drugs (13% vs. 17%, *p* = 0.004), and an overall 17% decrease in trauma admissions. There was also a significant increase in alcohol positive patients from Period 1 to Period 2 (32% vs. 39%, *p* = 0.007), with no change in drug positive patients (57% vs. 52%, *p* = 0.13).

**Table 1:** Overall Rates of Alcohol and Drug Screens and Use by COVID-19 Time Period, *N* = 4762

Characteristics, n (%)	Period 1, *N* = 2602 (55%)	Period 2, *N* = 2160 (45%)	*P*-value
Screened			
*Alcohol (missing 16)*	873 (34%)	790 (37%)	**0.03**
*Drug (missing 7)*	552 (21%)	560 (26%)	**0.001**
*Both*	342 (13%)	363 (17%)	**0.004**
Screening results			
*Alcohol positive*	281 (32%)	304 (39%)	**0.007**
*Drug positive*	312 (57%)	292 (52%)	0.13
*Both positive*	75 (22%)	89 (25%)	0.42
*Neither positive*	112 (33%)	130 (36%)	0.39

Period 1: March 16, 2019-May 31, 2019; Period 2: March 16, 2020-May 31, 2020

**Fig. 1:**
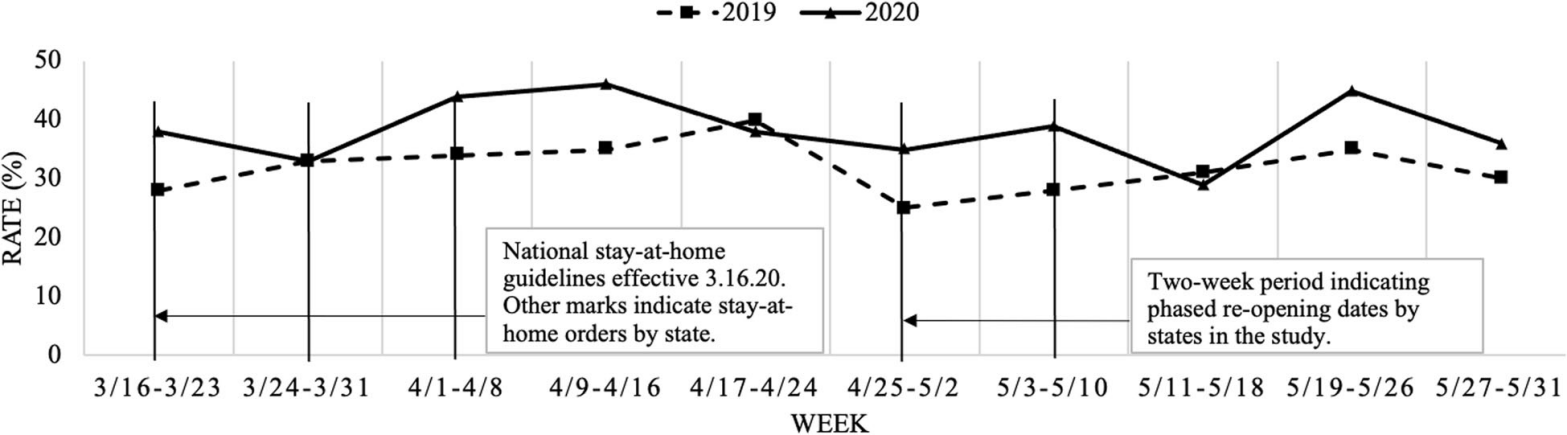
Alcohol Positive Rates by Week shows the rate of alcohol positive patients by year beginning with the week of the national stay-at-home guidelines on 3.16.20. The second and third marks indicate state-specific stay-at-home mandates, while the fourth and fifth mark indicate the start of state specific re-opening dates

**Fig. 2:**
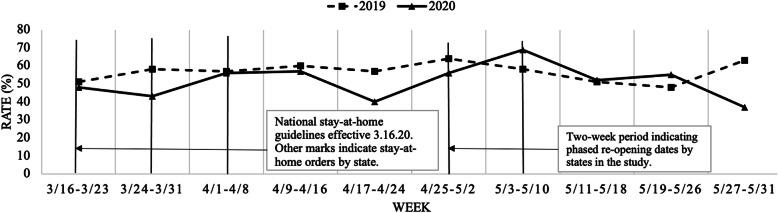
Drug Positive Rates by Week shows the rate of drug positive patients by year beginning with the week of the national stay-at-home guidelines on 3.16.20. The second and third marks indicate state-specific stay-at-home mandates, while the fourth and fifth mark indicate the start of state specific re-opening dates

Figure 1 shows rates of alcohol positive admissions by week and study period. Beginning with the national stay-at-home guidelines effective 3/16/2020, there was an immediate decrease within the first week, but in the following two weeks (3/24/20–4/8/20), the figure indicates a dramatic increase in alcohol positive rates that remained steady through 4/16/20. Following the first phased re-opening date on 4/27/20, there was another small increase and then a sharp decrease and increase, after which it leveled out with the previous years’ rates after 5/10/20. The rates by drug positive admissions over the same time periods (Fig. 2), follow a similar pattern to alcohol positive rates within the first two weeks, but rates are generally lower across time compared to 2019, and instead of a sharp increase after stay-at-home orders, the figure shows a sharp decrease.

Of the 4762 trauma patients admitted during Periods 1 and 2, 1025 (22%) were alcohol or drug positive (Table 2). Overall, these patients were overwhelmingly male (73%), white (66%), had a low to moderate ISS (1–15, 74%), with a median (IQR) age of 42 (29–49) years old, a median CCI of 0, and a median LOS of 3 (2–7) days. Fourteen percent of alcohol or drug positive patients also had a history of mental illness, 16% a history of alcoholism, and 13% a history of substance abuse. The top three most commonly used drugs included THC (31%), opiates (25%), and benzodiazepines (15%), and the median (IQR) BAC was moderately high at 142 mg/dL (35–233).

**Table 2:** Characteristics and Outcomes of Patients with Positive Alcohol or Drug Screening Results, *N* = 1025

Characteristics, n (%)	Period 1, *N* = 518 (50%)	Period 2, *N* = 507 (49%)	P-value
Sex, (male)	373 (72%)	371 (73%)	0.68
Race (white) (missing 58)			0.15
*White*	343 (71%)	329 (68%)	
*Black*	106 (22%)	109 (22%)	
*Other*	32 (7%)	49 (10%)	
Age range, *years*			0.44
*18–20*	21 (4%)	31 (6%)	
*21–34*	162 (32%)	152 (30%)	
*35–64*	244 (47%)	243 (48%)	
*≥ 65*	91 (18%)	81 (16%)	
Mechanism of injury			0.05
*Fall*	167 (32%)	147 (29%)	
*Motor vehicle crash*	132 (25%)	122 (24%)	
*Motorcycle crash*	39 (8%)	34 (7%)	
*Assault*	38 (7%)	22 (4%)	
*Gunshot wound*	51 (10%)	64 (13%)	
*Other*	91 (18%)	118 (23%)	
Injury severity score			0.37
*1–9*	297 (57%)	269 (53%)	
*10–15*	92 (18%)	96 (19%)	
*≥ 16*	129 (25%)	142 (28%)	
CCI, median (IQR), range	0 (0–2), (0–7)	0 (0–2), (0–7)	0.89
Comorbidities			
*Mental illness*	76 (15%)	67 (13%)	0.50
*Alcoholism*	75 (14%)	130 (26%)	**< 0.001**
*Substance abuse*	59 (11%)	115 (23%)	**< 0.001**
State			
*Colorado*	171 (33%)	160 (32%)	0.62
*Texas*	72 (14%)	98 (19%)	**0.02**
*Missouri*	187 (36%)	188 (37%)	0.74
*Kansas*	88 (17%)	61 (12%)	**0.02**
LOS, median (IQR), *days*	3 (2–7)	3 (2–7)	0.58
ICU stay	224 (43%)	192 (38%)	0.08
In-hospital mortality	23 (4%)	25 (5%)	0.70

Period 1: March 16, 2019-May 31, 2019; Period 2: March 16, 2020-May 31, 2020. CCI, Charlson Comorbidity Index; LOS, hospital length of stay; IQR, interquartile range; ICU, intensive care unit

In patients who were alcohol or drug positive, patient characteristics were similar by study period, with the exception of more patients in period 2 with a history of alcoholism (14% vs. 26%, *p* < 0.001, Table 2) and substance abuse (11% vs. 23%, p < 0.001). Additionally, there were significantly more alcohol or drug positive admissions during period 2 in Texas (14% vs. 19%, *p* = 0.02) and significantly fewer admission during period 2 in Kansas (17% vs. 12%, p = 0.02).

Of the drug positive patients, Methamphetamine use from Period 1 to Period 2 (2% vs. 0.2%, p = 0.02, Table 3) was significantly lower. There was also as non-significant increase in patients with a BAC ≥ 200 (very high) from Period 1 to Period 2 and a non-significant decrease in the range of the median number of drugs from (0–5) to (0–4). There were no other significant differences by alcohol and drug characteristics from Period 1 to Period 2.

**Table 3:** Drug and Alcohol Findings of Patients with Positive Alcohol or Drug Screening Results, N = 1025

Characteristics, n (%)	Period 1, N = 518 (50%)	Period 2, N = 507 (49%)	P-value
Drugs			
*Cocaine*	32 (6%)	27 (5%)	0.56
*Opiates*	130 (25%)	122 (24%)	0.70
*Benzodiazepines*	78 (15%)	75 (15%)	0.91
*Barbiturates*	8 (2%)	4 (0.8%)	0.26
*THC*	154 (30%)	161 (32%)	0.48
*Methamphetamine*	9 (2%)	1 (0.2%)	**0.02**
*Amphetamines*	64 (12%)	67 (13%)	0.68
*Ecstasy*	15 (6%)	10 (4%)	0.24
*PCP*	16 (3%)	17 (3%)	0.95
Number of drugs, median (IQR), range	1 (0–2), (0–5)	1 (0–2), (0–4)	0.65
BAC levels (mg/dL)			0.17
< 10	237 (46%)	203 (40%)	
10–79	64 (12%)	78 (15%)	
80–99	13 (3%)	12 (2%)	
100–199	99 (19%)	88 (17%)	
≥ 200	105 (20%)	126 (25%)	

Period 1: March 16, 2019-May 31, 2019; Period 2: March 16, 2020-May 31, 2020; THC, tetrahydrocannabinol; PCP, Phenylcyclohexyl piperidine; IQR, interquartile range; BAC, blood alcohol concentration; mg, milligram; dL, deciliter

## Discussion

This multicenter study examined the differences in alcohol and drug use among trauma patients admitted during the COVID-19 pandemic in 2020 as compared to the previous year. The study demonstrated increased screening for drugs and alcohol during the pandemic period and increased alcohol positive findings; of those who were positive for alcohol or drugs, there was a significant increase in SUDs in 2020 compared to the 2019 period.

Despite the challenges surrounding (trauma) patients with SUDs, particularly during the COVID-19 pandemic, to our knowledge this is one of the first studies examining how the pandemic and accompanying stay-at-home order affected substance use among multiple trauma centers. Most of the current studies on the effect of the stay-at-home orders on trauma patients have reported on changes in mechanism of injury, trauma volume, injury severity, and type of injury, and are typically single-center only studies. (Christey et al. 2020; DiFazio et al. 2020; Lubbe et al. 2020; Morris et al. 2020; Park et al. 2020; Rajput et al. 2020; Sutherland et al. 2020) Forrester and colleagues examined the impact of the shelter-in-place order on trauma activations from January–March 2020 to the same periods in 2018 and 2019 and reported the overall rate of alcohol (19%) and drug positive (11%) patients, but indicated there were no significant changes over time. (Forrester et al. 2020) In our study, the overall alcohol and drug positive rates were much higher at 35 and 23%, respectively, possibly due to examining the immediate time period after the start of the national stay-at-home order, while the former article examined overall rates across January–March 2018–2020. Leichtle et al. described the influence of the stay-at-home order on trauma volume and patterns from 3.17.2020–4.30.2020 to the same periods in 2018 and 2019 and in contrast to our study, found that that the rate of patients with alcohol intoxication (19.5% vs. 20.8%) and those with chronic substance abuse (7.3% vs. 9.7%) did not change, but similarly, the number of patients with chronic alcohol abuse significantly increased, while those with intoxication with other substance was unchanged. (Leichtle et al. 2020) It remains challenging to compare different patient populations across the country that also had different stay-at-home order dates. Across the four states in this study (CO, TX, MO, KS), there were four different stay-at-home order dates, as well as phased re-opening dates, (Kaiser Family Foundation 2020) potentially lending to the significant increases in alcohol or drug positive patients in Texas and significant decreases in Kansas, while other states remained unchanged.

Interestingly, although there was a significant increase in the rate of alcohol screens overall, we still observed a significant increase in the rate of alcohol positive patients. Substance use has long been associated with traumatic injury and with the global pandemic as an external stressor, higher use of alcohol and drugs may be expected, as was seen with other national disasters such as Hurricane Katrina and the September 11th terrorist attacks (Spagnolo et al. 2020). However, conversely, while drug screening rates increased from 2019 to 2020, there was a non-significant decrease in drug positive patients. The small decrease in drug positive patients may indicate a lack of access to drug supplies during the immediate period following the national stay-at-home orders as seen in Fig. 2. The current published literature has indicated an increase in self-reported binge-drinking or high-risk alcohol consumption during COVID-19; (Ahmed et al. 2020; The Telegraph 2020) however, we did not see a significant increase in the proportion of trauma patients positive for high BACs (≥200) from Period 1 to Period 2. (Morris et al. 2020; Sutherland et al. 2020) Other non-trauma population studies have reported increases in alcohol use: Aragona et al. examined the negative impacts of COVID-19 on mental health services for migrants in Europe and found that patients admitted with alcoholism increased over time from 2017 to 2020, (Aragona et al. 2020) while survey studies among various populations in the United States (≥18 years old) (Boschuetz et al. 2020; Lechner et al. 2020), China (≥14 years) (Ahmed et al. 2020), France (≥16 years old) (Rolland et al. 2020), and Poland (≥18 years old) (Chodkiewicz et al. 2020), identified increases in alcohol and drug use as well as alcohol use disorders during COVID-19 (Ahmed et al. 2020; Boschuetz et al. 2020; Chodkiewicz et al. 2020; Lechner et al. 2020; Rolland et al. 2020). Furthermore, the survey studies indicated that although there were small increases in alcohol use overall, the most significant shifts occurred in populations with a history of SUDs and mental illness, as this population frequently uses substances to cope with stressful situations (Boschuetz et al. 2020; Taylor et al. 2008).

The finding that significantly more patients were being admitted with SUDs is concerning and noteworthy. Patients with SUDs frequently present with specific medical risk factors, which make them more vulnerable to infections and other complications, (Spagnolo et al. 2020) but especially to COVID-19. Patients with SUDs have been shown to have a nearly 9-fold increased risk of COVID-19, as well as a significantly higher mortality, compared to patients without (Wang et al., 2021). During an already challenging hospitalization with COVID-19 protocols, patients with SUDs are at higher risk of increased complications, poor outcomes, and readmissions (Wang et al., 2021). Thus, trauma centers should be aware of the increase in patients with SUDs, in order to appropriately screen and manage this high-risk group; recognition of a patient’s substance use has been demonstrated to play an influential part in the provision of immediate medical care, referral to the appropriate secondary services, better acute pain management, and a decreased risk of further injury and death (Chung et al. 2019; Nicolson et al. 2014; Salottolo et al. 2019; Salottolo et al. 2017).

There are several study limitations. First, we defined drug positive patients as those with a positive urine drug screen at admission, which meant that patients who received opioids or other drugs prior to admission were potentially misclassified as positive. Second, because drug screen panels were not the same across each trauma center, several did not screen for PCPs, potentially underestimating the total drug categories reported. Third, there are no guidelines for drug and alcohol screening, the rate of testing was trauma center specific and the true rate of trauma patients positive for alcohol or drugs is probably underestimated; however, the use of trauma registry data did allow for a large and more diverse population, including all trauma patients admitted to six Level 1 trauma centers across four states. Fourth, COVID-19 testing results were not tracked at all participating centers and testing criteria varied over time across centers. Fifth, each state had different start and stop dates for the stay-at-home order, which may have contributed to variation in trauma patterns and volume by state; however, we believe the White House’s national date captured these trends and provided consistency across the study. Sixth, although the differences in screening rates were statistically significantly different by study arm, the differences were small and may not be clinically meaningful.

## Conclusions

The results of this study demonstrate that the COVID-19 pandemic and its accompanying stay-at-home mandates were associated with a significant increase in trauma patients admitted with a positive alcohol screen and in patients with substance use disorders. As ICUs continue to grow with the second wave of the COVID-19 pandemic, it is pertinent that trauma centers continue to target higher risk patient populations, such as patients with a history of substance use disorders, in order to best prioritize and manage care.

## Data Availability

Because of agreements with the Institutional Review Boards overseeing this study, original deidentified datasets from the trauma registries at the six participating sites are not publicly available. Some limited analysis datasets and SAS code used to conduct statistical analyses may be available, upon reasonable request to DBO.

## Abbreviations

COVID-19: Corona Virus Disease 2019
SARSCoV-2: Severe acute respiratory syndrome, Corona Virus, 2
PTSD: Post-traumatic stress disorder
SUDs: Substance use disorders
BAC: Blood alcohol concentration
UDS: Urine drug screen
PCP: Phenylcyclohexyl piperidine
THC: Tetrahydrocannabinol
ISS: Injury severity score
LOS: Hospital length of stay
ICU: Intensive care unit
GSW: Gunshot wound
MVC: Motor vehicle crash
MCC: Motorcycle crash
CCI: Charlson Comorbidity Index
IQR: Interquartile range
